# A pilot study of scanning acoustic microscopy as a tool for measuring arterial stiffness in aortic biopsies

**DOI:** 10.1016/j.artres.2015.11.001

**Published:** 2016-03

**Authors:** Riaz Akhtar, J. Kennedy Cruickshank, Xuegen Zhao, Brian Derby, Thomas Weber

**Affiliations:** a Centre for Materials and Structures, School of Engineering, University of Liverpool, L69 3GH, UK; b Diabetes & Cardiovascular Medicine, Nutritional Sciences Division, King's College London, Franklin Wilkins Building, 150 Stamford Street, London SE1 9NH, UK; c School of Materials, The University of Manchester, Manchester M13 9PL, UK; d Cardiology Department, Klinikum Wels-Grieskirchen, Grieskirchnerstrasse 42, 4600 Wels, Austria

**Keywords:** Arterial stiffening, Scanning acoustic microscopy, Cholesterol, Glucose, Blood pressure, Pulse wave velocity, Acoustic wave speed

## Abstract

This study explores the use of scanning acoustic microscopy (SAM) as a potential tool for characterisation of arterial stiffness using aortic biopsies. SAM data is presented for human tissue collected during aortic bypass graft surgery for multi-vessel coronary artery disease. Acoustic wave speed as determined by SAM was compared to clinical data for the patients namely, pulse wave velocity (PWV), blood pressure, cholesterol and glucose levels. There was no obvious trend relating acoustic wave speed to PWV values, and an inverse relationship was found between systolic and diastolic blood pressure and acoustic wave speed. However, in patients with a higher cholesterol or glucose level, the acoustic wave speed increased. A more detailed investigation is needed to relate SAM data to clinical measurements.

## Highlights

• Scanning acoustic microscopy (SAM) is a potential tool for arterial stiffness.
• SAM provides a measure of the acoustic wave speed.
• In this pilot study, no clear trend was observed with pulse wave velocity.
• Blood pressure was inversely related with acoustic wave speed.
• Trends observed with other clinical markers such as glucose and total cholesterol.

## Introduction

Scanning acoustic microscopy (SAM) is a useful tool for determining the mechanical properties of vascular tissue.^1^, ^2^, ^3^, ^4^, ^5^, ^6^ The image contrast in a SAM derives from differences in the propagation speed of acoustic waves (typically with a frequency of 100 MHz–1 GHz) through a material; this wave speed is related to the elastic properties of the material, in particular Young's modulus.^7^ The advantage of SAM over other in vitro mechanical property characterisation techniques for vascular tissue is that it is an imaging method.^8^ The contrast in an acoustic image in the frequency range 100 MHz–1 GHz is comparable to that obtained from optical microscopy and is sufficient to resolve distinct features such as collagen fibres. Obtaining both histological and mechanical information^9^ should be useful for studies on arterial stiffening, given the structural changes in collagen and elastin as the main load-bearing components of (large) arteries.^10^ Therapies have been proposed which aim to target the assembly, degradation or stiffness of elastic fibres in large arteries^11^ and hence may be ideal to determine the efficacy of such treatments. An additional advantage is that SAM can be applied to small biopsy samples, for example <1 cm^2^ of tissue.

In our previous work, we used SAM to examine age-related changes in an ovine model of ageing. There we found that the acoustic wave speed, as determined by SAM, increased with age, and that the increased acoustic wave speed was localised to the inter-lamellar region of the medial lamellar unit in the aorta.^10^ We also used SAM as a tool to examine the role of a hypertrophy-inducing cytokine, Cardiotrophin 1 (CT-1), on vascular stiffening in a rat model^12^ and in an experimental model of diabetes in rats.^5^ In CT-1 treated rats, we reported an increase in acoustic wave speed in the aorta, matching *in vivo* mechanical property measurements. In the diabetic model, we also found profound changes in the aorta with diabetes could be localised to the inter-lamellar region of the vessel wall and linked to increased extracellular protease activity.^5^

SAM has previously been used for characterisation of human aortic tissue, for example to examine atherosclerosis, the stress distribution in atherosclerotic plaques, and aortic aneurysms.^1^, ^2^, ^3^ Here, we showcase some pilot data collected from human aortic biopsies and explore the use of the technique as a tool in human arterial stiffening, we believe for the first time.

## Methods

Aortic biopsy samples were obtained from 8 patients undergoing aortic bypass graft surgery for multi-vessel coronary artery disease at the cardiothoracic surgery department, Klinikum Wels-Grieskirchen, Austria. The study was approved by our regional ethics committee (Ethics committee of Upper Austria, EK number E-2-10), and all patients gave written informed consent. Details for the patients are presented in Table 1.

The biopsy samples were prepared for cryosectioning by freezing in optimal cutting temperature (OCT) resin (Sakura Fintek Europe BV, Alphen aan den Rijn, The Netherlands) in pre-cooled isopentane and stored at −80 °C. The OCT resin is water-soluble and does not affect the mechanical properties of the tissue. The frozen tissue blocks were subsequently sectioned to nominal 5 μm thickness sections. The sections were imaged with SAM (SAM2000 microscope, PVA TePla Analytical Systems GmbH, Herborn, Germany) as has been reported in detail previously with the Multi-Layer Phase Analysis (MLPA) method.^13^ This allows the determination of the acoustic wave velocity from each pixel in the image. Imaging was conducted at 760 MHz which provided a nominal spatial resolution of ∼1.3 μm.

**Figure 1:**
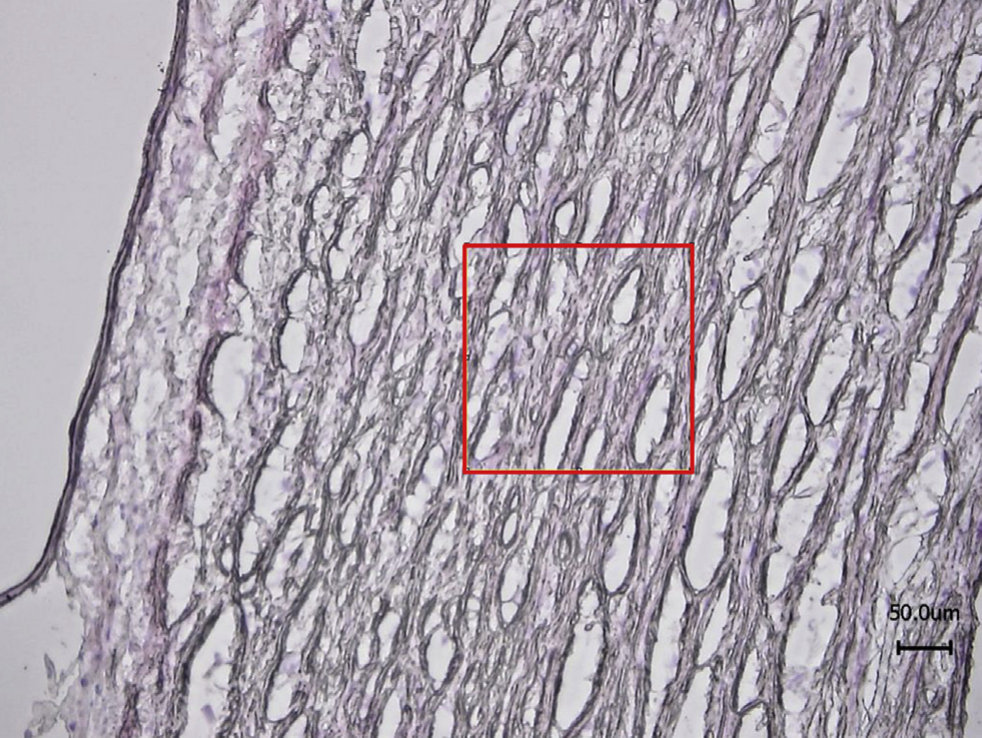
Typical H&E image of aorta with a 200 × 200 μm region of interest marked in the media for SAM imaging. Note, the serial sections used for SAM did not require any staining.

A series of 200 × 200 μm images were captured with the SAM and the mean acoustic wave speed was determined from each image.^13^ Each image compromised 512 × 512 pixels and three locations in the media were measured for each sample. Hence, the mean acoustic wave speed was averaged from 786,432 pixel measurements for each sample. For each sample, serial sections were stained with Haemotoxylin and Eosin (H&E) and imaged with optical microscopy (Nikon Eclipse 50i, Surrey, UK) to locate the medial region of the vessel for subsequent imaging with SAM (Fig. 1).

Pearson's correlation coefficient was used for all statistical analysis with the following parameters presented: Pearson's r. adjusted r^2^ and p-values for each group. All statistical analysis was conducted with OriginPro 9 (OriginLab Corporation, Northampton, MA, USA).

## Results

**Figure 2:**
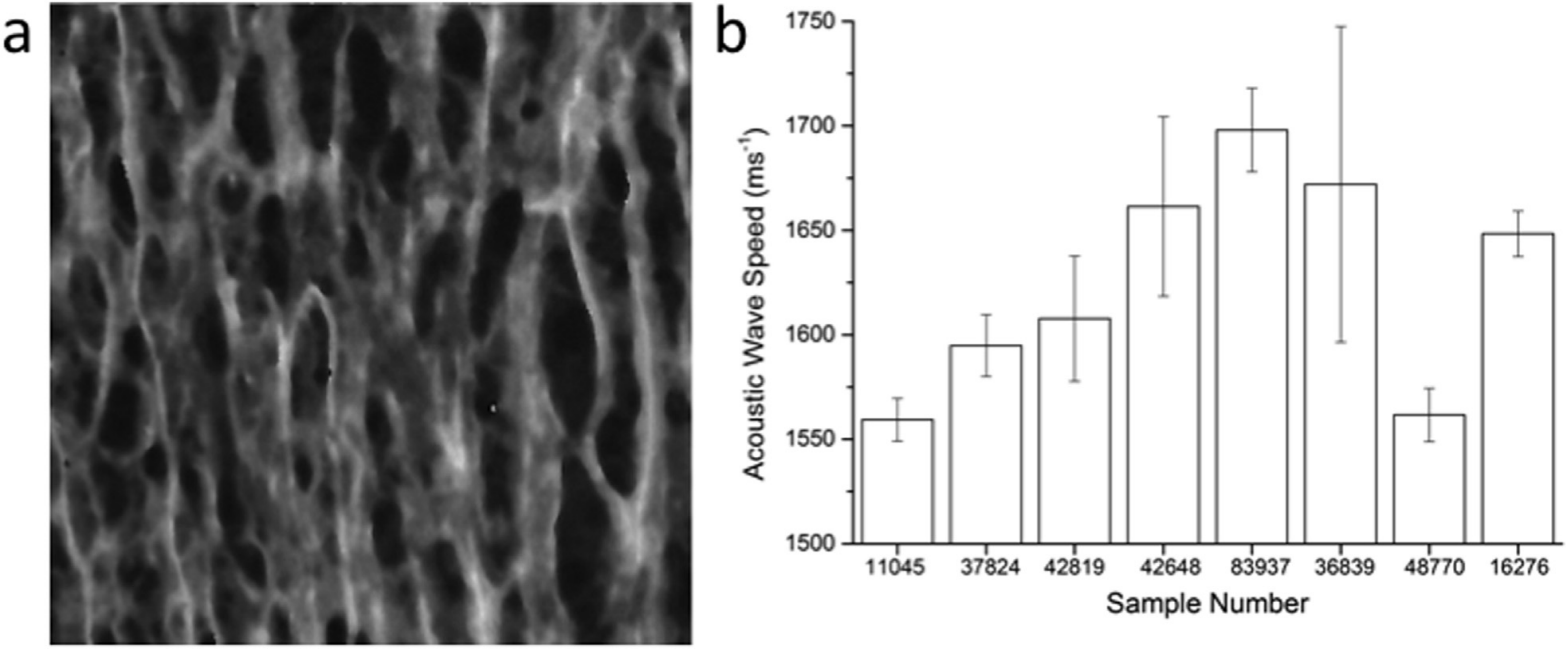
a) 200 × 200 μm SAM image of the medial layer for Sample 37824; b) Mean values for acoustic wave speed as determined by SAM for the different samples tested. Error bars represent standard deviation.

A typical SAM image is shown in Fig. 2a. The SAM data not only allows extraction of quantitative data (Fig. 2b) from the images but also allows qualitative assessment of the biopsy sample from the histological information, which is clearly visible (Fig. 2a).

The range of acoustic wave speed values determined in this study (approximately 1500–1700 ms^−1^) matched those reported by Saijo et al.^1^

The SAM data were plotted firstly against invasive, catheter-based measures of PWV and BP, and then against blood concentrations of total cholesterol and glucose in the patients.

**Figure 3:**
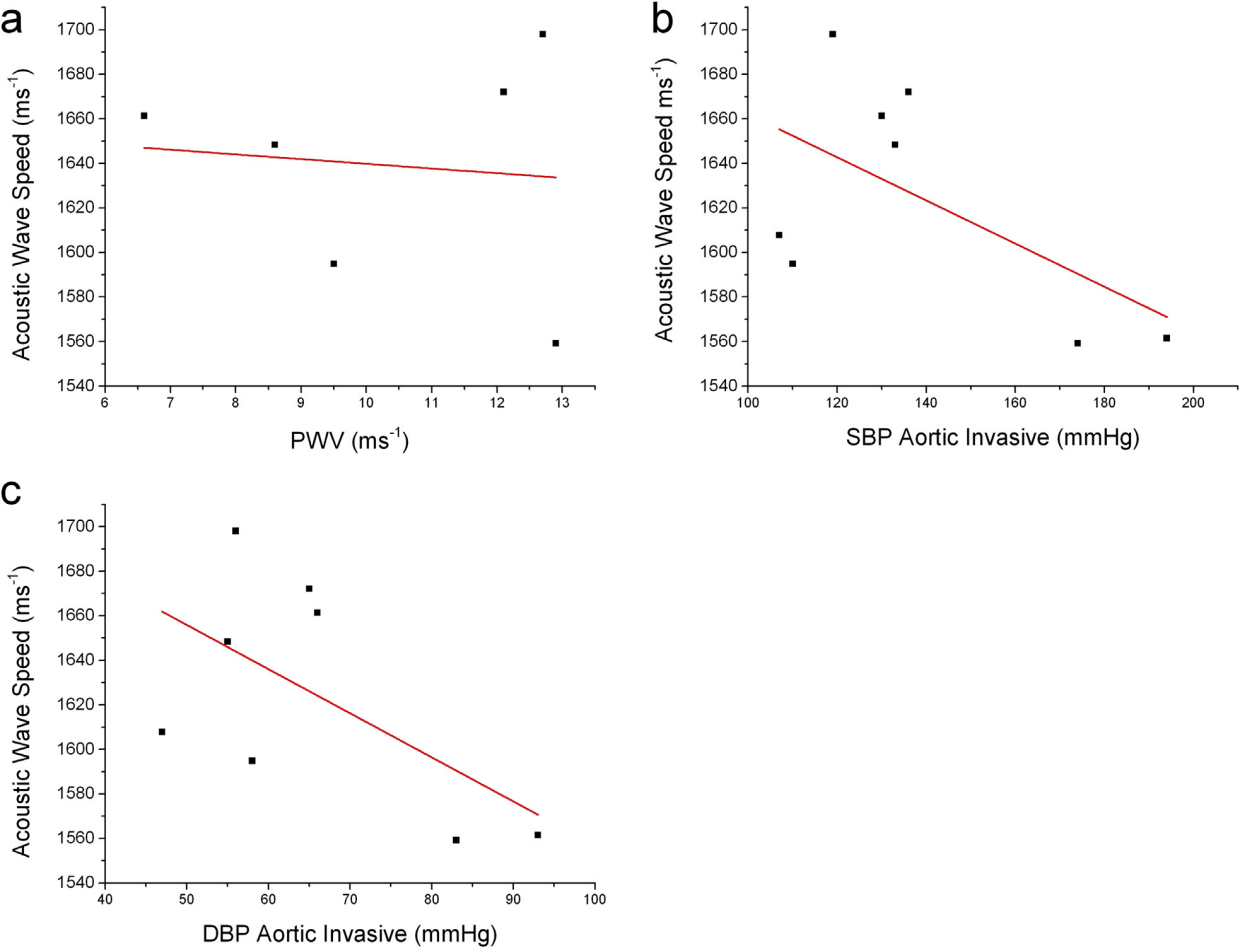
a) PWV measured during angiography vs vitro Acoustic Wave Speed (AWS), r = −0.1, r^2^ = −0.24 p = 0.84; b) Aortic SBP during angiography vs. AWS, r = −0.59, r^2^ = 0.22, p = 0.14; c) Invasive aortic DBP vs AWS, r = −0.59, r^2^ = 0.23, p = 0.13.

In this small sample study, PWV values were not obviously related to acoustic wave speed (Fig. 3a), while invasive BPs were, inversely (r = 0.22 systolic, 0.23 diastolic), with p values 0.13–0.14, (Fig. 3b and c). It should be noted that PWV data were only available for 6 of the patients hence the total sample size was 25% smaller than the other variables that are presented in this study.

**Figure 4:**
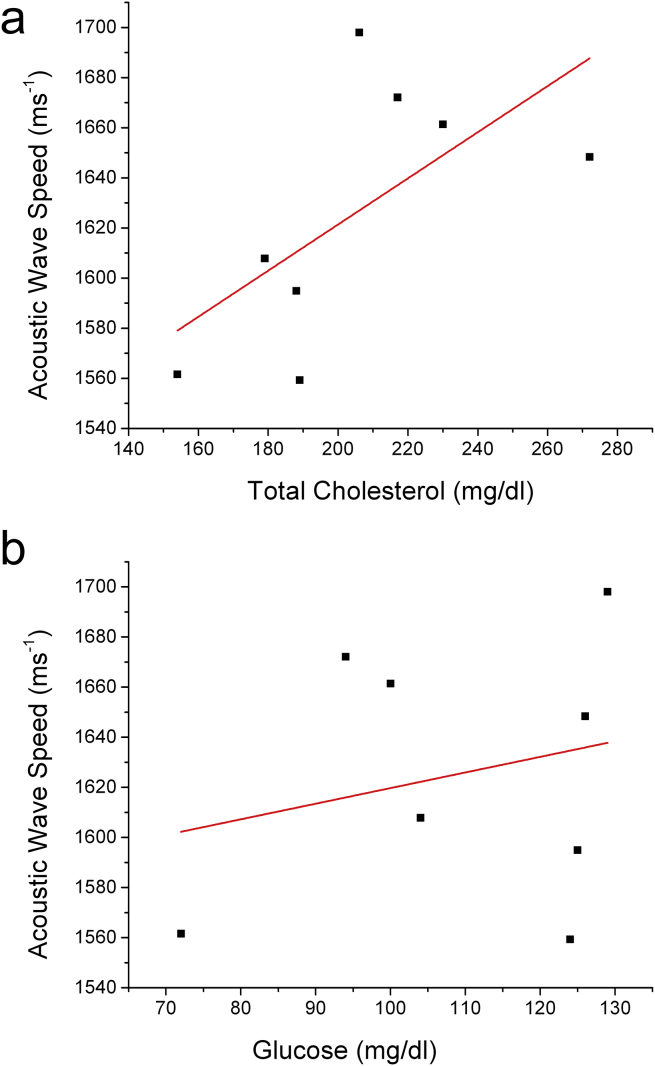
a) Total cholesterol, r = 0.64, r^2^ = 0.31, p = 0.09; b) Plasma glucose, r = 0.24, r^2^ = −0.1, p = 0.56.

Total cholesterol and plasma glucose values tended to be higher with higher acoustic wave speeds (Fig. 4a and b).

## Discussion

This pilot study explores the utility of SAM as a tool for characterisation of arterial stiffening in aortic biopsies. SAM has been used previously to study atherosclerosis^1^, ^2^ and aneurysms^3^ in human aortas. However, to our knowledge there are no previous studies which have used SAM in relation to arterial stiffening in human tissue. Our previous work focussed on animal tissues^5^, ^8^, ^10^, ^12^. Here, we examined the potential use of SAM for understanding human arterial stiffness. There was no correlation with PWV data, not surprising given the small numbers. Here, only localised regions in the medial layer of the aorta are examined by SAM, whereas the great clinical advantage of aortic PWV is its make-up from all parts of the aortic wall including the adventitia. It is highly likely that “classical” aortic PWV is a net effect of the mechanical properties of many components across the longitudinal, cylindrical and cross-sectional dimensions of the aortic wall. Hence PWV is providing a measurement at the whole organ level whereas SAM gives micro-detail for specific regions of the aortic wall. Acoustic wave speed ex-vivo here investigates the contributions of the different layers of the vessel separately. The wave speed measured with SAM is 1000 higher than “classical” aortic PWV, due to that fundamental difference. PWV is a measure of the rate at which pressure waves propagate along the vessel. In contrast, SAM measures the speed of sound in materials. PWV is correlated to the physical properties of the tissue through the Moens-Korteweg equation (Equation (1)):

(1) $$PWV=\sqrt{\frac{Et}{2r\rho }}$$

where *E* is the mean elastic modulus of the arterial wall, *ρ* is the mean density of the blood, *t* is the thickness of the vessel wall and *r* is its radius; the ratio *t/r* is assumed constant along the measurement length.

Acoustic wave speed (c) as determined by SAM is related to the physical properties of the tissue, as its stiffness, through Eq. (2):

(2) $$c=\sqrt{\frac{E\left(1-\nu \right)}{\rho \left(1+\nu \right)\left(1-2\nu \right)}}$$

where E is the tissue Young's modulus, ρ is the tissue density and ν is the Poisson's ratio. At 1 GHz, the spatial resolution of the technique is around 1 μm.

In the Moens-Korteweg equation there are a number of inherent assumptions, which mean it cannot be used to make a robust measurement of mean elastic modulus of the arterial wall in an individual.^14^ However, in the clinical setting PWV is easy to measure^15^ and is considered to be both reproducible^16^ and a strong predictor of cardiovascular events and all-cause mortality.^17^ In contrast, SAM provides highly localised data and does not represent the *in vivo* state of the entire aortic wall.

Counter-intuitively, we found an inverse relationship between SBP and acoustic wave speed. This is surprising given that blood pressure plays a significant role in determining changes in the aortic wall structure^18^ However, this is most likely related to the small sample size with only one single measurement of blood pressure. The negative relationship with DBP would be expected, as in this elderly group of individuals, an increase in aortic stiffness is associated with a decrease in DBP. However, the problem with sample size and single blood pressure value applies to DBP as well. Furthermore, we noted that the correlation of SBP and DBP with PWV in this data are also weak (r^2^ = −0.07 and r^2^ = −0.11).

The systemic effect of elevated cholesterol and glucose in blood appears to be associated with measureable changes in the localised mechanical properties of the medial layer with SAM. The mechanisms linking arterial stiffness with high cholesterol and glucose are related to enzymatic degradation of elastin,^5^, ^19^ which may explain the relationship here with these parameters and micromechanical properties in the medial layer. A more substantive study would be required to investigate the relationship between these parameters further in human tissue biopsies.

If SAM is to become more widely used, a number of technical challenges will apply. For example, because high spatial resolution techniques such as SAM are highly sensitive to sample preservation techniques, surgeons will require access to facilities to orientate the samples carefully, before embedding them in the appropriate medium and rapidly freezing at −80 °C. Such sample preparation is of paramount performance given that the structural integrity of the aortic tissue is highly affected by preservation technique.^20^ However, the ability to be able to target stiffness measurements to very specific components of the aortic wall is appealing for many applications.

In conclusion, SAM is a useful tool for characterizing small volumes of aortic biopsy tissue. A more substantive study is needed to test whether SAM data are related to clinical measurements of arterial stiffness.

## Conflict of interest statement

There are no conflicts of interests to declare.

## Figures and Tables

**Table 1:** Selected characteristics of the subjects; BMI (Body Mass Index); HT (Hypertension); SAM AWS (Acoustic Wave Speed); S (systolic), D (diastolic) BP (Blood Pressure); Heart Rate (HR); PWV (Pulse Wave Velocity).

Sample	Sex	Age years	BMI kg/m^2^	HT	Diabetes	SAM AWS m/s	SBP aortic invasive mm Hg	DBP aortic invasive mm Hg	HR	Total cholesterol mg/dl	Glucose mg/dl	Creatinine mg/dl	PWV aortic invasive m/s
11045	m	73	26.3	Yes	Yes	1558	174	83	94	189	124	1.5	12.9
37824	m	76	23.6	Yes	Yes	1587	110	58	70	188	125	1.3	9.5
42819	m	80	28.7	Yes	No	1608	107	47	57	179	104	2	
42648	m	59	28.7	No	No	1651	130	66	55	230	100	1.3	6.6
83937	m	80	26.5	No	No	1709	119	56	91	206	129	1.1	12.7
36839	m	66	26.8	Yes	No	1616	136	65	91	217	94	1.5	12.1
48770	f	71	30.8	Yes	No	1538	194	93	79	154	72	0.8	
16276	f	70	37.8	Yes	Yes	1637	133	55	72	272	126	1	8.6
